# An Insight into the Molecular Characteristics and Associated Pathology of Chicken Astroviruses

**DOI:** 10.3390/v14040722

**Published:** 2022-03-30

**Authors:** Abdullahi Abdullahi Raji, Abdul Rahman Omar

**Affiliations:** 1 Laboratory of Vaccine and Biomolecules, Institute of Bioscience, Universiti Putra Malaysia (UPM), Serdang 43400, Malaysia; abdullahi.raji@udusok.edu.ng; 2 Department of Veterinary Pathology, Faculty of Veterinary Medicine, Usmanu Danfodiyo University, Sokoto 840212, Nigeria; 3 Department of Veterinary Pathology and Microbiology, Faculty of Veterinary Medicine, Universiti Putra Malaysia (UPM), Serdang 43400, Malaysia

**Keywords:** chicken astrovirus, diversity, similarity, pathology

## Abstract

The chicken astrovirus (CAstV) is a ubiquitous enteric RNA virus that has been associated mainly with conditions, such as the runting-stunting syndrome, severe kidney disease, visceral gout, and white chick syndrome, in broiler-type chickens worldwide. Sequence analysis of the capsid genes’ amino acids of the strains involved in these conditions reveals a genetic relationship and diversity between and within the CAstV genogroups and subgroups based on phylogenetic analysis, genetic distance (p-dist), and pathogenicity. While the two genogroups (A and B) are demarcated phylogenetically, their pairwise amino acid sequence identity is 39% to 42% at a p-dist of 0.59 to 0.62. Group-A consists of three subgroups (Ai, Aii, and Aiii) with an inter- and intra-subgroup amino acid identity of 78% to 82% and 92% to 100%, respectively, and a p-dist of 0.18 to 0.22. On the other hand, the six subgroups (Bi, Bii, Biii, Biv, Bv, and Bvi) in Group-B, with a p-dist of 0.07 to 0.18, have an inter- and intra-subgroup amino acid identity of 82% to 93% and 93% to 100%, respectively. However, these groupings have little to no effect on determining the type of CAstV-associated pathology in chickens.

## 1. Introduction

Undoubtedly, for over five decades, the poultry industry has witnessed rapid growth, contributing significantly to the provision of animal protein and creating a global niche in food economics. Driven by the growing demand for poultry meat and eggs worldwide, it is projected that the industry will continue to witness a modest pace of growth for the next 10 years [1]. However, emerging and re-emerging poultry viral pathogens continue to be of great concern to the industry worldwide [2]. Among these viral pathogens are enteroviruses and those formerly classified as entero-like viruses or small round viruses (SRVs), which are isolated from chickens with growth retardation, general under-performance, and mortality [3]. Typically based on electron microscope morphologic presentation, SRVs could fall into Caliciviridae, Piconaviridae, Circoviridae, Parvoviridae, or Astroviridae. Of these five viral families, Astroviridae has been reported to cause diarrhea, enteritis, and other related illnesses and conditions in humans and several mammalian and avian species [4,5]. 

Known to be among the leading causative pathogens of diarrhea in birds and mammals, and ubiquitous in both sick and healthy humans, animals, and birds, astroviruses were first discovered in 1975 during an electron microscopic (EM) examination of diarrheic stool samples from young children of 2 years old and below that were suffering from gastroenteritis [6]. Astroviruses are now a pervasive enteric virus worldwide and a leading cause of enteritis and diarrhea in neonates, the immunocompromised, and the aged. Shortly after their discovery, small round virus particles with astrovirus morphology were recorded in the early 1980s in domestic animals, particularly calves and lambs with gastroenteritis [7,8] and ducks with viral hepatitis, leading to acute mortality [9]. These observations conceivably served as the earliest indication of extra-intestinal tropism of the astrovirus [10]. Interestingly, in the 1960s, a similar report recognized the astrovirus as the etiologic agent of duck hepatitis [11]. Over the years, a wide range of animal species have been found to be susceptible to astroviral infections, including synanthropic and domesticated animals and avian and mammalian species on the land and in the water. Although astroviruses continue to be among the least-studied viruses, the virus has been mentioned in several articles since its first detection in humans in the 1970s. In this review, we discuss our recent understanding of the molecular characteristics and pathogenicity of the chicken astrovirus (CAstV).

Presently, the two recognized and characterized astroviruses of economic importance in the poultry industry are the avian nephritis virus (ANV) and chicken astrovirus (CAstV) [12,13]. These viruses are serologically different and antigenically similar to one another. Chicken astrovirus was first identified in 2004 from a cultured sample of two broiler flocks exhibiting uneven growth and the runting-stunting syndrome (RSS) in the Netherlands [13]. Analysis of the 320-bp reverse transcription (RT)-PCR product of the RNA-dependent RNA polymerase (RdRp) genes, generated from RNA recovered from an isolate, revealed that CAstV is related to the turkey astrovirus (TAstV) and ANV but is totally different from them and earlier identified poultry astroviruses [13]. Some studies on the structural characteristics of CAstV before 2004 had led to its misdiagnosis and misidentification as the small round virus (SRV) or entero-like virus (ELV) of the Piconaviridae [14]. Studies have associated CAstV with enteritis, RSS, and uneven growth [13,15], and kidney disease with severe visceral gout in young broiler chickens [16]. The virus has also been identified as the cause of the “white chick” syndrome or hatchery disease, with a high affinity to young chicks and susceptible naïve in-lay breeder chickens at the peak of their production period [17,18,19,20].

## 2. Astrovirus

### 2.1. Virus Structure

The name astrovirus originates from the ultrastructural morphological presentation of the virus under the electron microscope with five spikes of a star (astron in Greek) [21]. It is worth noting that the star appearance depends on the pH and will not be present in more than 10% of the infectious particles. The assembly of the virus particles is from an approximately 90-kDa product of the VP90 capsid precursor protein, which further undergoes cellular caspase processing, leading to the generation of VP70 protein, while losing an acidic terminal. The breakage of caspases is an essential component in the release of the virion; thus, the C-terminal 8-kDa caspase cleavage component is strongly retained across genotypes [22], hence indicating its role in replication [23]. Through extracellular trypsin-like proteases, the latent infectious virus particles are again transformed into three viral proteins (VP34, VP27, and VP25) to form an active virion [24]. The three viral proteins then become the core of the capsid (VP34) and the spike domains (VP27 and VP25).

Generally, astrovirus virion sizes under the electron microscope (EM) are within the range of 28 to 35 nm in diameter [6,25]. The virion is up to 41 nm in size when propagated in monolayer cell cultures, while exhibiting a much larger appearance with conspicuous protruding spikes without star-like features [26]. Similarly, alkaline treatment of samples enhances the star characteristics of the virus. The viruses are small and round with smooth unbroken edges and triangular hollowed surfaces that are white centered [6]. However, the source of the astrovirus and the processing of the samples for EM could alter the size of the virus [27]. The virus particles have generally a T = 3 icosahedral symmetry.

### 2.2. Genome Organization

**Figure 1:**
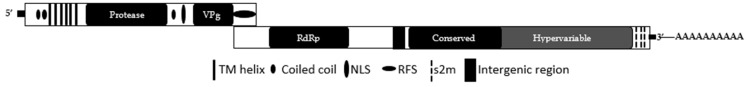
Genomic organization of CAstV. TM helix, Transmembrane helix; NLS, Nuclear localization signal; RFS, Ribosomal frameshift; s2m, Stem-loop II-like motif; VPg, viral protein genome linked; RdRp, RNA dependent RNA-polymerase and AAAAA, polyadenylated tail.

The genome of chicken astrovirus is similar to that of other astroviruses and consists of a linear, positive-sense single-stranded (ss) RNA (+ssRNA) with a length that varies from 7 to 7.6 kb without the polyadenylated (poly-A) tail at the 3′-end. The genome is composed of three serially arranged genes in the form of open reading frames (ORFs): *ORF-1a*, *ORF-1b*, and *ORF-2*, which are bound by untranslated regions (UTRs) on both the 5′- and 3′-ends [28]. The 5′-end region is the shortest of all known avian astrovirus 5′-end regions [29]. The first two ORFs (*ORF-1a* and *ORF-1b*), upstream of the genome, are composed of non-structural proteins (NSPs): trypsin-like serine protease, a viral protein genome-linked (VPg) at the end of the 5′-end possessing a TEEEY-like residue (SEEEY), and RNA-dependent RNA polymerase (RdRp) genes, which are all responsible for replication and virion generation [30] (Figure 1).

These two NSPs overlap with a nucleotide sequence with a length of between 12 and 45 nucleotides (nt) in avian astroviruses and contain a heptanucleotide “slippery sequence” (5′-AAAAAAC-3′) region, which is part of a ribosomal frameshift structure similar to the frameshift structures found in retroviruses and coronaviruses [31,32]. Putatively, *ORF-1a* encodes a bipartite nuclear localization signal (NLS), six possible transmembrane (TM) domains, a coiled-coil (CC) domain, and a helicase (HEL) domain [18,30] (Figure 1). The NLS is a feature found in CAstVs, HAstVs, and OAstVs but not in TAstV. Notably, *ORF-1b* has a start codon (ATG), which makes it a true ORF by definition; it is highly conserved and has a consistent length in CAstVs [29]. With this, it is proposed that the translation of *ORF-1b* in CAstV starts through a start codon near the 5′-end of the heptanucleotide region [29]. The third and last ORF (*ORF-2*) encodes a structural protein and a viral capsid protein precursor that is expressed from a sub-genomic (sg) RNA, and it is situated at the 3′-end of the astrovirus genome. The region is documented to be within a range of 670 to 744 amino acids (aa) in length across various avian astroviruses [33]. In fact, *ORF-2* of all astroviruses encodes a sizeable polyprotein that generates capsid protein when cleaved by cellular protease. The capsid protein gene has been studied extensively; much of what is currently known about the astroviruses, and their relatedness, comes from its analyses. The capsid protein has three significant domains: the RNA-binding, N-terminal, and C-terminal domains. The N-terminal domain is more conserved than the RNA-binding domain and the C-terminal domain. The N-terminal domain contains a preserved motif of repeats, SRSRSRSRSRSR, and highly basic residues similar to those of DAstV and TAstv-2 [33]. As in many RNA viruses, the region participates in the encapsidation of astroviruses and virion formation [34].

The 5′-end and 3′-end untranslated regions (UTRs) exhibit lengths similar to those of other sequenced bird astroviruses. In comparison, the 5′-end UTR of CAstV is between 7 and 21 nt, while the 5′-end UTRs of TAstV and DAstV are 10 and 23 nt, respectively. The 3′-end UTR is between 200 and 282 nt, which is within the range of ANV with 305 nt and TAstV with 192 nt [29]. A coronavirus stem-loop II-like motif (s2m), which exists in some RNA viruses, such as astroviruses, picornaviruses, coronaviruses, and caliciviruses from avian and mammalian species, is present at the 3′-end UTR of CAstV [35] (Figure 1). In a recent study, we observed that CAstVs possess two highly conserved s2ms that are composed of 43 nt and spaced by 56 nt at the 3′-end UTR, as well as one or two highly conserved s2ms. Interestingly, although we observed this presentation in all the Group-B CAstVs available in GenBank, three Malaysian isolates (MT491730, MT491731, and MT491732] and two Chinese isolates (MN725025 and MN725026) of the same Group-B exhibited an additional s2m, while the Polish CAstV isolate (KT886453), the only characterized complete genome in Group-A CAstV, has a single s2m [30]. 

Generally, mutations within the s2ms restore base pairing via several compensatory mutations. The extensive conservation of this motif across many viruses indicates that the region plays a vital role in the replication of positive-sense RNA viruses [35]. Although no precise function has been identified, s2m presumably influences the virus’s proteins and the host’s cellular proteins during RNA replication [36]. However, it is yet to be determined whether the presence of the third s2m in the Malaysian and Chinese isolates can influence the genomic activities of the isolates. Similarly, the s2m is a highly conserved region in Astroviridae but is reportedly missing in AstV-MLB1 and rat, bat, and turkey astroviruses [36,37]. While the absence of s2m suggests that it is a non-essential feature in avastroviruses, it has been proposed that the region evolved as a result of recombination with non-related viruses [38]. Biologically, the significance of the absence of or variations in s2m or ORF arrangement is uncertain but could indicate changes due to evolutionary pressure [33].

It is worth noting that CAstV *ORF-1b* and *ORF-2* are in the +1-reading frame of *ORF-1a*, thus resembling the genome arrangement of TAstV-1 (Figure 1). On the whole, there exist variations in the length of the ORFs across species and serotypes. However, *ORF-1b* consistently appears to be the least divergent, while *ORF-2* is the most divergent because of the region’s hypervariability and exposure to selective pressure [10].

### 2.3. Classification and Characterization

In 1995, the International Committee on Taxonomy of Viruses (ICTV) recognized the Astroviridae family based on a consistent phylogenetic reconstruction that divided it into two genera: the genus Mamastrovirus and the genus Avastrovirus, infecting mammalian and avian species, respectively [26,27]. However, since 2008, this classification has become more complex due to an increase in the number of animal species (covering no fewer than 30 mammalian species and 14 avian species). Moreover, characterization-based sequencing has revealed inadequacies in the above classification criteria by demonstrating that viruses could be genetically alike even when isolated from diverse hosts and could simultaneously display a broad assortment of sequences within an individual host type [27]. For this reason, a decade ago, the Astroviridae study group proposed that the standardization of astrovirus classification be based on genetic differences in the capsid gene amino acid sequences encoded in the *ORF-2* region of the virus [27], with viruses exhibiting a greater distance than approximately 0.30 to 0.35 regarded as separate genotype species. On average, the mean amino acid distance (p-dist) between MAstV and AAstV is 0.83, while it is 0.72 within the MAstV genus and 0.704 ± 0.013 within the AAstV genus. Similarly, the ICTV has proposed a p-dist of 0.576 to 0.741 between Avastrovirus genotypes and a p-dist of 0.204 to 0.284 within the genotypes [18,26]. However, in CAstVs, the p-dist between the two recognized genotypes (Group-A and Group-B) is 0.59 to 0.61, and the p-dist is 0.01 to 0.61 within Group-B, indicating lower p-dist values than those proposed by the ICTV (Table S1).

Presently, the ICTV recognizes 19 species of mamastrovirus (MAstV-1 to MAstV-19) belonging to two major groups: genogroup I and genogroup II, with roughly 14 new strains awaiting grouping, of which some are tentatively identified as species [39,40]. On the other hand, the Avastrovirus genus consists of astroviruses characterized from domestic birds (poultry) and ornamental, wild, and aquatic aviary species. An earlier classification recognized only the turkey astrovirus-1 (TAstV-1) in Avastrovirus genogroup I (GI.A), avian nephritis virus 1 and 2 (ANV-1 and ANV-2) in Avastrovirus genogroup I (GI.B), and TAstV-2 and duck astrovirus (DAstV) in Avastrovirus genogroup II (GII.A) [26,41]. However, these classifications are now referred to as Avastrovirus-1 (AAstV-1), Avastrovirus-2 (AAstV-2), and Avastrovirus-3 (AAstV-3) for GI.A, GI.B, and GII.A, respectively [26,41]. Four strains of Avastrovirus, including CAstV, await inclusion into the Avastroviridae family, in addition to the three existing Avastrovirus genogroups (Avastrovirus-1 to Avastrovirus-3). 

Thus, as usual for most avian diseases, records on the classification or variety of viruses most often emanate from poultry studies due to an understandable lack of evidence from wild species [42,43]. Remarkably, the Astroviridae currently constitute an emerging and genetic diverse group with many host species that would require regular updates.

Currently, in line with the above, the diversity and classification of CAstVs is based on phylogenetic analysis and the genetic distance (p-dist) between *ORF-2* (capsid) gene amino acid sequences [44] (Table S1 and Figure S1). Similarly, Todd in 2009 reported a widespread antibody against the two serogroups, with a subsequent genotyping study confirming the clustering of the CAstV strains into CAstV Group-A and Group-B. However, before this, in the early 1990s, two distinctive serogroups of CAstV were recognized based on the degree of cross-reactivity with a heterologous antiserum [14,45].

These genotyping and strain clustering results correspond to the amino acid identity shared across the capsid gene encoded in *ORF-2* at a lower level of 38% to 40% [44,46]. Group-A CAstV consists of three subgroups (Ai, Aii, and Aiii), with inter-subgroup homologies ranging from 77% to 82%, and four subgroups (Bi, Bii, Biii, and Biv), with shared inter-subgroup identities of 84% to 85% within the B group of CAstV [44]. Accordingly, the intra-subgroup percentage amino acid identity within Group-B CAstV is 94% to 100%, except for subgroup Bii, which has an amino acid identity share of 92% to 97% because of incomplete capsid sequences of isolate 4175 (Table S1). 

In 2021, Raji et al. isolated and characterized three unique Malaysian isolates (IBS503/2017, IBS543/2017, and UPM1019/2018), which shared a 100% amino acid capsid identity. The three isolates clustered and formed a new subgroup (Bv) in Group-B CAstV based on the earlier described share of inter- and intra-identity [30]. The isolates were from three broiler flocks within Peninsular Malaysia, exhibiting clinical signs and lesions that included uneven growth and performance, enlarged kidneys, urate deposits, and visceral gout [30]. The three isolates share an identity of 37% to 39% with members of Group-A CAstV, while sharing an inter-subgroup amino acid identity of 85% to 91% with Group-B CAstV (Table S1). Additionally, in a recent metagenomic study in the Netherlands, the three characterized strains (Chicken_V_M_038_astro_4, Chicken_V_M_046_astro_12, and Chicken_V_M_047_astro_4) were genetically related to Group-B CAstVs, with an identity share of 80% to 88% [47] (Table S1 and Figure S1). Thus, suggesting an additional subgroup Bvi within Group-B CAstV is in line with its intra-subgroup identity share of 98% to 100%. 

Therefore, conveniently, except for the members of subgroups Bii and Ai, a capsid gene amino acid sequence identity percentage of ≥94% and a p-dist of 0.00 to 0.04 to a reference or prototype strain indicate the emergence of a distinct variant or subgroup within the CAstV genogroups.

Currently, there are 347 sequences of CAstV deposited in GenBank, of which approximately 9% (30) are for near-complete or complete genomes, approximately 58% (200) are for partial *ORF-1b* sequences (which are mostly used in the initial detection of the virus), and approximately 34% (117) are for the partial or complete capsid gene. This limited number (117) of capsid gene sequences poses some difficulties for classification and the detection of recombination events, especially with the emergence of new incomplete capsid sequences. Similarly, events between different types of CAstVs may lead to variations in host or tissue tropism and are more difficult to investigate when only partial genes or genomes are used, as most recombination processes do not occur exclusively in antigenic protein-encoding regions of astrovirus genes but rather in the complete genome [48].

## 3. Chicken Astrovirus Infection

### 3.1. Transmission

Chicken astrovirus (CAstV) is a relatively recently emerged virus that is originally associated with malabsorption syndrome, which includes RSS in broiler chickens, uneven performance in growth, and enteritis in flocks. It is also a ubiquitous virus that is commonly isolated from the gut of clinically diseased and healthy chickens [13,25,49]. Several investigators have reported the abundance of astrovirus in the intestines of broiler chickens and its prevalence in commercial chickens and turkey flocks from the ages of 2 to 6 weeks [49,50,51]. The abundance of astrovirus, especially in broiler chickens, occurs mostly between the ages of 2 to 4 weeks, which happens to be the prime time of enteritis [49]. However, other factors, such as variation in the CAstV isolate pathogenicity, the quantity of the infectious virus, the presence and interactions with other infectious enteric viral pathogens, and the maternal antibody levels, might play a significant role in the manifestation of clinical symptoms, hindering the attainment of optimum performance [49].

As a member of the RNA enteric viruses [49], CAstV usually infects chickens at an early age [4,13,25,49]. The fecal-oral route is the major transmission route [32]. Reports have demonstrated that young chickens are mostly infected horizontally via contaminated housing due to an inadequate downtime period between flocks of either broilers or layers and a weak biosecurity that could lead to the introduction of the virus into the farm [4]. Similarly, some evidence indicates possible vertical transmission of CAstV from naïve in-lay parents to embryos, with hatched chicks shedding the virus early in life [4,52,53]. These excreted viruses will then be transmitted horizontally to susceptible chicken mates [4,32].

Like other known astroviruses, CAstV infects mostly young animals of commercially reared chickens and turkeys [4,54,55]. The virus primarily infects the broiler-type of chicken, which happens to be the type of bird the virus was first isolated from in [13]. Reports have demonstrated that CAstV is prevalent among the broilers’ great-grandparents, grandparents, parents, and the commercial flocks of broiler chickens [46,56]. A seroprevalence survey has also demonstrated that the CAstV antibody is widely spread among the parents of commercial laying chickens and layer flocks [57,58,59] and in indigenous or local breeds of chickens [57,58]. Experimental infections demonstrate that 1-day-old SPF chickens are equally susceptible to CAstV infection [4,14,16,18,30,46,60,61,62] (Table 1). While mature chickens present subclinical to relatively mild clinical signs, they shed the virus into the litter and transfer it vertically to young chickens [46].

Permissive and susceptible young chickens within the first 10 days of age exhibit varying illnesses because of different degrees of infection. The observable clinical signs consist of diarrhea, maldigestion, growth retardation, or mild runting, while severe clinical signs can lead to death after infection [13,15,55]. This highlights the wide virulence range of the virus under field conditions, with clinical signs varying from runting to death as a result of malabsorption, nephropathy, and visceral gout [16,64,65]. Usually, clinical signs set in as early as 3 to 4 days post infection (dpi) in the form of diarrhea, but normally, the signs are noticeable in birds that are 6 to 12 days old and could last for 21 days. There are noticeable traces of uneven and stunted birds, often typified by fecal stripes and pasted vents [13,66], as well as other signs, such as dullness, mild to moderate depression, anorexia, increased culling rate, huddling, irregular feathering, leg weakness, and anorexia [13]. Generally, in less severe cases, the clinical signs last for only 10 days.

### 3.2. CAstV-Associated Clinical Illnesses or Conditions

Presently, three to four types of CAstV-related illnesses or conditions exist based on infectious virus strains [4]: RSS or uneven growth [13,25], enteric and locomotion disorders [55], kidney disease with visceral gout [16], and white chick hatchery disease [17] or white chick syndrome (WCS) [2,67,68].

#### 3.2.1. Uneven Flock Performance and the Runting-Stunting Syndrome (RSS)

Identified in the broiler industry in the 1970s as a transmissible disease of broiler chickens of doubtful etiology [15,69,70,71], RSS affects very young broiler chickens with characteristic signs that include diarrhea, stunting, and ruffled feathers, leading to significant economic deficits, especially in the broiler industry across the globe [15,25,72]. Several enteric and entero-like viruses have been implicated as the cause of growth issues in chickens with the capability of causing retardation and uneven flock performance [4,14,37,49,62,70,71,73,74]. Although these enteroviruses are associated with growth checks and retardation, not all have been reported to cause RSS in an experimental study. However, reoviruses, parvoviruses, coronaviruses, and astroviruses are known to be the agents of well-defined illnesses and have been demonstrated to cause retardation following oral inoculation in 1-day-old chickens [12,25,45,71,74,75]. As the definitive etiological agent of RSS is still being debated, it appears that an infectious agent is involved and that there is the likely involvement of an RNA virus, considering the varying degree of pathogenicity and retardation in 1-day-old broiler chickens and specific-pathogen-free (SPF) chickens inoculated with CAstV isolates [14,76]. Similarly, CAstV isolate inoculations have not produced the severe growth checks typical of RSS, where young broiler chicks may exhibit less than half of their predicted bodyweight at 21 to 28 days old, implying that other agents or variables are possibly at play [4]. The syndrome has several synonyms according to its clinical presentation, including the malabsorption syndrome, brittle bone disease, helicopter disease, and infectious stunting syndrome [65]. 

Compared with their cage mates, clinically affected birds exhibit ruffled feathering and growth retardation [25,29]. Additionally, close to 20% of the infected flock exhibited retardation in growth (dwarfing or stunting), with some chickens weighing only 30% to 50% of their healthy cage mates, huddling for warmth, and a >50% increased culling rate as a result of widespread uneven growth [4]. The Malaysian strain (UPM1019/2018), belonging to the newly formed Bv subgroup of the Group-B CAstV, was documented to cause a dramatic growth retardation of 20% in infected and exposed sentinel 1-day-old SPF chickens on day 9 post inoculation [30]. However, a 70% retardation by 12 dpi has been reported by Kang et al. [25] in a study on broiler chickens. This conspicuous variation in weight retardation is mostly observed when broiler chickens are used in experimental studies because of the fast developmental growth rate of broiler chickens. Exhibiting all the clinical signs associated with RSS, the isolate further expresses the capability of infecting naïve exposed young chickens (sentinels) during experimental infection.

Generally, intestinal lesions are strain dependent based on experimental infection. Isolate 612 (Group-A CAstV (Ai)) of South Africa [60] and UPM1019/2018 (Group-B CAstV (Bv)) of Malaysia [30] are mild in pathogenicity and relatively similar when compared with the FP3 (Group-B CAstV (Bi)) of the UK, which were reported to cause severe intestinal lesions as early as 1 day post inoculation and to alter the villous-to-crypt ratio at 3 and 6 dpi. Similarly, changes are equally observed in the gut, causing either decreased secretion of enzymes that help digest feed or alterations that could lead to the blockage of absorption of nutrients [32,65,77,78]. The affected chickens have pale, distended intestines filled with mucoid intestinal contents. Microscopically, notably reported lesions, although not pathognomonic to CAstV infection, are atrophy of intestinal villi and cysts in the crypts of Lieberkühn [25,29,65].

Recently, metagenomic findings have demonstrated that aside from being ubiquitous in healthy broiler chickens, RNA viruses in birds have a community composition that continues to diversify as the birds age [49]. Nevertheless, with disease severity linked to CAstV genomic variations, an in-depth analysis of CAstV illnesses, especially genome sequencing based on next-generation sequencing (NGS), would be an essential tool for establishing the relationship between strain variations and the severity of disease or illness [4].

#### 3.2.2. Severe Kidney Disease, Urate Deposits, and Visceral Gout

With a diverse origin that includes infectious bronchitis virus (IBV) and management and nutritional factors [75], severe outbreaks of kidney disease and visceral gout have also been associated with certain CAstV strains circulating in India, the Middle East, and, recently, Malaysia [4,16,30,79]. In India, a mortality rate of 40% was recorded in an outbreak that affected commercial broiler flocks from 2011 to 2012 [16]. Although ANV was initially linked to the outbreak, molecular investigations ruled out IBV, ANV, management and nutritional factors [16]. The infected broiler chickens exhibited clinical signs that included enlarged urate-filled ureters with visceral and articular gout [16]. 

As with most other CAstV infections, clinical signs are overall dullness, drowsiness, and diarrhea within 3 to 4 dpi in very young broilers and in experimentally infected 1-day-old SPF chickens [16,30,79]. Experimental infections with the Indian strains (the PDRCs in the north, south, west, and east), initially referred to as the Indian subgroup Bi but presently Biii in Group-B CAstV, produced a mortality of 67.5% to 100% in commercial broiler and SPF chickens, respectively [16]. Between 2017 and 2018, outbreaks with kidney disease and visceral gout were recorded in most commercial broiler flocks in Peninsular Malaysia. However, the incriminating agent was a CAstV strain that had an identity share of between 87% and 89% with the Indian strains (Biii) and FP3 (Bi) [30]. Although the virulent Malaysian isolate (UPM1019/2018) of the subgroup Bv of Group-B CAstV was comparatively milder than the Indian strain and FP3, it presented similar lesions that included a white chalky covering on the heart, a white thin membranous covering on the liver, and the enlargement of the ureters and kidneys, with both containing urates [4,16,30]. A 7% mortality rate increase was recorded in the SPF experimental study, against the mortalities reported in the Indian strain trial study. In experimentally infected SPF-ECEs, visible lesions are marked embryo dwarfing, a pale and necrotic liver, and enlarged kidneys. Histopathologically, renal tubular congestion and degeneration, as well as parenchymatous interstitial nephritis with extensive urate deposits, were observed. Lymphocytic infiltration and crystal formation in the kidneys were lesions expressed by the Indian, UK, and Malaysian strains with varying degrees of severity [16,30,79].

Consequently, the extensive variety of circulating CAstV strains in the UK, India, the Middle East, and Europe, causing severe renal pathology leading to gouty lesions, and the level of *ORF-2* amino acid similarity, especially between the Indian and Middle East strains, strongly support the involvement of CAstV. In line with this premise, CAstV should equally be considered in the list of differentials in cases of kidney disease and gout in chickens. Similarly, based on the lesions observed in broiler flocks across Malaysia between 2017 and 2018, and with the subsequent chicken trial with CAstV isolate UPM1019/2018, there exists palpable evidence for the nephropathogenic potential of the circulating Malaysian CAstV strains [30]. 

#### 3.2.3. White Chick Syndrome or White Chick Hatchery Disease

For over 40 years, CAstV has been associated with a hatchability condition referred to as “white chick” [20,68]. The condition, which is mostly observed in 1-day-old broiler chicks, is typified with overall pale plumage, irregular curly brown feathers on the neck region extending to the head, and green to bronze discolored liver lesions [2,17,20,53,67,68]. Additionally, the observable signs and lesions in white chick syndrome (WCS) are similar to those in RSS, including abnormal feathering, runting, underperformance, wet cloaca, weakness, and mortality within the first few hours and up to 3–5 days after hatching [2,17,20,68].

A temporary but significant decrease of 4% to 68% in hatchability, and mid to late deaths of in-shell embryos, have been reported in broiler breeder flocks in Finland and Poland [68]. Similar episodes have been identified in commercial broiler breeder flocks in the US, UK, Canada, Norway, and Brazil [4,18,53,67,68]. Classically, the CAstV strain that causes WCS affects breeder flocks between the ages of 30 and 40 weeks and often with a temporary egg drop [68].

Histopathologic lesions may include bile duct proliferation, cholangiofibrosis, cholestasis, hepatic necrosis with glycogen accumulation, a rise in hepatic extramedullary granulopoiesis, and heterophilic and lymphocytic interstitial nephritis [2,20,67].

In line with Koch’s postulate, the condition has been reproduced by inoculating embryonated chicken eggs, leading to associated hatching problems and a high mortality rate, with deaths recorded a few hours after hatching at a 100% mortality rate [67,68,80]. Chicks hatched alive exhibit characteristic features of WCS [18]. Similarly, WCS CAstV strains cause no pathological alterations in the intestine, but reduced nutrient absorption occurs as a result of osmotic diarrhea [81,82].

Presently, strains belonging to the Aiii of Polish origin and members of the Biv subgroup that includes strains from Brazil and Canada [4,18,53] have been associated with WCS despite group variation.

## 4. Control and Prevention

Based on serological profiling, it is possible to produce and keep birds free of astrovirus diseases. However, the ubiquity of avian astroviruses in domestic birds suggests that eradicating the virus from birds, particularly commercial poultry, is challenging given the virus’s ability to be transmitted vertically [32]. In addition, astroviruses are resistant to a wide range of disinfectants, and their persistence in the environment makes eradication a monumental task. Strict biosecurity, enhanced timing, an extended interval between production cycles, and sufficient cleaning and decontamination of the environment will help reduce the risk of the avian astrovirus infection [32].

Poultry wastes, including poultry droppings, must be collected and removed on a regular basis in such a way that passages within the poultry facilities are free of contamination. The virus is efficiently and effectively removed by peroxymonosulfate (MPS) product, Virkon-S 1.5%, 0.3% formaldehyde, 0.1% propriolactone, and 90% methanol [32].

Multiple age farming should be avoided, as it prolongs low performance by allowing infected hens to recover without showing clinical manifestations but yet shedding the virus [31].

There are currently no prophylactic drugs or vaccines available to treat and prevent CAstV in poultry. The transfer of adequate maternal antibodies from breeder hens to chicken embryos is critical for limiting vertical virus transmission and providing timely defense against horizontal virus transmission. Although the exact age of the initial infection is unknown, the severity of the sickness is determined by the age of the first exposure; therefore, timely immunity is recommended [4]. It has been proposed that an efficient vaccination will protect and provide immunity to hens against the genotypically and serologically diverse CAstV strains presently in circulation [4]. Consistent activation of the CAstV-specific antibody in three- and four-weeks post-inoculation in SPF-chicks suggests protection [76]. As the field-type exposure of breeder hens to the CAstV strain of the white chick syndrome tends to provide a lifelong resistance, it is expected that a similar longevity could be achieved via vaccines, but not without empirical confirmation.

## 5. Conclusions

Compared with other infectious avian viruses, namely the avian influenza virus, avian coronavirus, fowl adenovirus, and avian paramyxovirus, there exists a dearth of information on the impact of CAstV on the poultry industry. A key factor for this has largely been due to the lack of standardized propagation systems of either cell culture or animal model that will allow biological characterization. Furthermore, genetic diversity, type of chicken affected, and the variations in clinical disease caused by the different strains of the CAstV complicate the capacity to develop a solid story similar to that of other members of Astroviridae. However, in the face of these challenges, over the past 15 years, significant progress has been made. Of all the avian astroviruses, CAstV is second only to human astroviruses in terms of characterization, thus allowing the understanding of the virus’s pathogenic mechanism and immunity characteristics. Yet, more research is needed.

Importantly, there is an urgent need to develop a standard protocol for naming and grouping existing and future genus members. A standard method for comparing molecular characteristics would improve the understanding of the virus relationships within genera and identify potential virulence determinants. In trying to better understand CAstV, the avian virology community should try to characterize representative members of each genogroup, and subgroup fully. This would entail the development of a sequencing procedure, as well as the development of antibodies and other reagents. All these studies would contribute to a better understanding of the cellular, physiological, and immunological alterations CAstV causes in their host species so that we can develop more effective prevention, control, and therapeutic strategies.

## Figures and Tables

**Table 1:** In vitro and in vivo pathogenicity study of different subgroups of CAstV strains.

No.	Accession Number	Isolate	Group/Subgroup	Isolate Origin	Isolation of Virus	Changes in SPF-Embryonated Chicken Egg and Cell Line	Clinical and Pathological Findings in Chicken	Reference
1	JN582328	FP3	Bi	Meconium of dead-in-shell embryo	Yolk sac of SPF embryonated chicken eggs	Stunted embryo growth	Shrunken degenerate cells and nuclear debris in the epithelium of many villi; Multifocal cellular infiltration of the renal interstitium and scattered necrotic tubules	[60,63]
2	MK746105	CHN/2017/NJ1701	Bi	Small intestine of “Yellow” chickens with mild growth problems	LMH and yolk sac of SPF embryonated chicken eggs	CPE detection of virus antigens; stunted embryo; significant decrease in hatching rate		[61]
3	MW846319	GD202013	Bii	Kidneys and intestine	Yolk sac of embryonated chicken eggs	Stunted embryo	Enteritis, swollen kidneys; abscission in small intestines; necrotic and dilated renal tubules filled and infiltrated with red blood cells and heterophils	[52]
4	KY038163	ANAND/2016	Biii	Spleen, kidneys, and lungs of broiler chickens with visceral gout	Allantoic fluid of SPF embryonated chicken eggs	Embryo death within 5 to 6 days post inoculation; Hemorrhages on embryos		[62]
5	JX945861	PDRC (Indian isolates)	Biii	Kidneys from broiler flocks with severe mortality within first week of hatch	CEKC: and allantoic fluid of SPF embryonated chicken eggs	CPE: Stunting and yellowish discoloration of embryo	Dullness, drowsiness; 67.5 to 100% mortality in SPF and broiler chickens, respectively; necrosis of the liver; pale swollen kidneys with urate deposits, visceral and articular gout; tubular congestion, parenchymatous interstitial nephritis, and lymphocyte infiltration in the kidneys	[16]
6	KX397575	CC_CkAstV	Biv	Intestines of chickens with RSS	LMH and yolk sac of SPF embryonated chicken eggs	Retarded growth	Cystic lesions in the intestinal (duodenum) crypts	[25]
7	MT491731	UPM1019/2018	Bv	Kidneys of broiler chickens with gout and urate deposit		Stunted and hemorrhagic embryo, with mortality	Diarrhea, drowsiness, ruffled feathers, retarded growth, and depression. Air-filled empty crop with distended intestines; swollen kidneys, enlarged ureters with extensive urate deposits and visceral gout; heart covered with white chalky urate deposit	[30]
8	JN582317	612	Ai	From broilers with respiratory distress	CAM of SPF embryonated chicken eggs	Pock-like lesions and generalized edematous thickening of CAM: Stunted and hemorrhagic embryos.	Slight mottling of liver and mild swelling of kidneys; very mild lesions in the intestines; shrunken degenerate cells in the crypts and cell debris in the lumen; multifocal randomly distributed mononuclear cell infiltrates with degenerate hepatocytes; mild to moderate mononuclear cell and occasionally heterophilic infiltration of the interstitium and necrotic tubules in the kidneys	[14,60]
9	JN582318	P22.18.8.00	Ai	From broiler chicks with RSS	CEL and LMH	CPE and plaque production	Mild diarrhea, partly digested feed in the feces; distention of the small intestine; small areas of limited damage at the base of the villi of the small intestine	[13]
10	KT886453	G059/2014	Aiii	Liver and kidneys of dead in shell embryos, and weak chicks with white plumage	CAM and yolk- sac of SPF embryonated chicken eggs	Delay and prolong hatching; weak chicks; growth inhibition and white chicks	Visible subcutaneous edema; greenish exudate in the body cavity; enlarged and congested liver with brittle consistency and numerous pinpoint hemorrhages on the surface; kidneys, spleens, and pancreas were enlarged and very congested	[18]

Key: SPF; specific-pathogen-free: CAM; chorioallantoic membrane: CPE; cytopathic effect: CEKC; chicken embryo kidney cells: CEL; chicken embryo liver: LMH; hepatocellular carcinoma epithelial cell line.

## Data Availability

Not applicable.

## Supplementary Materials

The following supporting information can be downloaded at https://www.mdpi.com/article/10.3390/v14040722/s1. Figure S1: phylogenetic tree of CAstV. Using capsid gene amino acid sequences, the evolutionary history was inferred using the neighbor joining (NJ) method. Trees were constructed using 1000 bootstrapped replicates in MEGA-X [83]. Branch lengths represent the number of substitutions per site. Table S1: evolutionary divergence of CAstV strains.

## References

[1] Global Poultry Production to Reach 137M Tonnes in 2020, Mainly Driven by Growth in China, the EU, and the UK. https://www.globaltrademag.com/global-poultry-production-to-reach-137m-tonnes-in-2020-mainly-driven-by-growth-in-china-the-eu-and-the-uk/.

[2] Saif Y.M., Swayne D.E., Pantin-Jackwood M.J., Spackman E., Johnson T.J., Day J.M., French D., Gingerich E., Bilgili S.F., Jones K., Swayne D.E., Boulianne M., Logue C.M., McDougald L.R., Nair V., Suarez D.L., de Wit S., Grimes T., Johnson D., Kromm M. (2020). Emerging Diseases and Diseases of Complex or Unknown Etiology. Diseases of Poultry.

[3] Cortez V., Meliopoulos V.A., Karlsson E.A., Hargest V., Johnson C., Schultz-Cherry S. (2017). Astrovirus biology and pathogenesis. Annu. Rev. Virol..

[4] Smyth V.J. (2017). A review of the strain diversity and pathogenesis of chicken astrovirus. Viruses.

[5] Iturriza-Gómara M., Cunliffe N.A., Ryan E.T., Hill D.R., Solomon T., Aronson N.E., Endy T.P. (2020). 34—Viral Gastroenteritis. Hunter’s Tropical Medicine and Emerging Infectious Diseases.

[6] Madeley C.R., Cosgrove B.P. (1975). 28nm particles in faeces in infantile gastrornteritis. Lancet.

[7] Snodgrass D.R., Angus K.W., Gray E.W., Menzies J.D., Paul G. (1979). Pathogenesis of diarrhoea caused by astrovirus infections in lambs. Arch. Virol..

[8] Woode G.N., Gourley N.E., Pohlenz J.F., Liebler E.M., Mathews S.L., Hutchinson M.P. (1985). Serotypes of bovine astrovirus. J. Clin. Microbiol..

[9] Gough R.E., Borland E.D., Keymer I.F., Stuart J.C. (1985). An outbreak of duck hepatitis yype II in commercial ducks. Avian Pathol..

[10] De Benedictis P., Schultz-Cherry S., Burnham A., Cattoli G. (2011). Astrovirus infections in humans and animals—Molecular biology, genetic diversity, and interspecies transmissions. Infect. Genet. Evol..

[11] Asplin F.D. (1965). Duck hepatitis: Vaccination against two serological types. Vet. Rec..

[12] Imada T., Yamaguchi S., Mase M., Tsukamoto K., Kubo M., Morooka A. (2000). Avian nephritis virus (ANV) as a new member of the family astroviridae and construction of infectious ANV cDNA. J. Virol..

[13] Baxendale W., Mebatsion T. (2004). The isolation and characterisation of astroviruses from chickens. Avian Pathol..

[14] McNeilly F., Connor T.J., Calvert V.M., Smyth J.A., Curran W.L., Morley A.J., Thompson D., Singh S., Mcferran J.B., Adair B.M. (1994). Studies on a new enterovirus-like virus isolated from chickens. Avian Pathol..

[15] Kang K., El-gazzar M., Sellers H.S., Dorea F., Williams S.M., Kim T., Collett S., Mundt E. (2012). Investigation into the aetiology of runting and stunting syndrome in chickens. Avian Pathol..

[16] Bulbule N.R., Mandakhalikar K.D., Kapgate S.S., Deshmukh V.V., Schat K.A., Chawak M.M. (2013). Role of chicken astrovirus as a causative agent of gout in commercial broilers in India. Avian Pathol..

[17] Sajewicz-Krukowska J., Pać K., Lisowska A., Pikuła A., Minta Z., Króliczewska B., Domańska-Blicharz K. (2016). Astrovirus-induced “white chicks” condition—Field observation, virus detection and preliminary characterization. Avian Pathol..

[18] Sajewicz-Krukowska J., Domanska-Blicharz K. (2016). Nearly full-length genome sequence of a novel astrovirus isolated from chickens with ‘white chicks’ condition. Arch. Virol..

[19] Jacukowicz A., Domańska-Blicharz K. (2017). Astrowirusy u drobiu. Med. Weter..

[20] Long K.E., Hastie G.M., Ojkić D., Brash M.L. (2017). Economic impacts of white chick syndrome in Ontario, Canada. Avian Dis..

[21] Caul E.O., Appleton H. (1982). The electron microscopical and physical characteristics of small round human fecal viruses: An interim scheme for classification. J. Med. Virol..

[22] Arias C.F., Dubois R.M. (2017). The astrovirus capsid: A review. Viruses.

[23] del Rocío Banos-Lara M., Méndez E. (2010). Role of individual caspases induced by astrovirus on the processing of its structural protein and its release from the cell through a non-lytic mechanism. Virology.

[24] Mendez E., Salas-Ocampo E., Arias C.F. (2004). Caspases mediate processing of the capsid precursor and cell release of human astroviruses. J. Virol..

[25] Kang K.-I., Linnemann E., Icard A.H., Durairaj V., Mundt E., Sellers H.S. (2018). Chicken astrovirus as an aetiological agent of runting-stunting syndrome in broiler chickens. J. Gen. Virol..

[26] Bosch A., Guix S., Krishna N.K., Méndez E., Monroe S.S., Pantin-Jackwood M., Schultz-Cherry S., King A.M.Q., Lefkowitz E., Adams M.J., Carstens E.B. (2012). Family—Astroviridae. Ninth Report of the International Committee on Taxonomy of Viruses.

[27] Bosch A., Pintó R.M., Guix S. (2014). Human astroviruses. Clin. Microbiol. Rev..

[28] Mendez E., Arias C.F., Knipe D.M., Howley P.M. (2007). Astroviruses. Fields Virology.

[29] Kang K.-I., Icard A.H., Linnemann E., Sellers H.S., Mundt E. (2012). Determination of the full length sequence of a chicken astrovirus suggests a different replication mechanism. Virus Genes.

[30] Raji A.A., Ideris A., Bejo M.H., Omar A.R. (2022). Molecular characterisation and pathogenicity of novel Malaysian chicken astrovirus isolates. Avian Pathol..

[31] Koci M.D., Seal B.S., Schultz-Cherry S. (2000). Molecular characterization of an avian astrovirus. J. Virol..

[32] Koci M.D., Schultz-Cherry S. (2002). Avian astroviruses. Avian Pathol..

[33] Pantin-Jackwood M.J., Strother K.O., Mundt E., Zsak L., Day J.M., Spackman E. (2011). Molecular characterization of avian astroviruses. Arch. Virol..

[34] Krishna N.K. (2005). Identification of structural domains involved in astrovirus capsid biology. Viral Immunol..

[35] Kofstad T., Jonassen C.M. (2011). Screening of feral and wood pigeons for viruses harbouring a conserved mobile viral element: Characterization of novel astroviruses and picornaviruses. PLoS ONE.

[36] Finkbeiner S.R., Kirkwood C.D., Wang D. (2008). Complete genome sequence of a highly divergent astrovirus isolated from a child with acute diarrhea. Virol. J..

[37] Pantin-Jackwood M.J., Spackman E., Woolcock P.R. (2006). Molecular characterization and typing of chicken and turkey astroviruses circulating in the United States: Implications for diagnostics. Avian Dis..

[38] Monceyron C., Grinde B., Jonassen T. (1997). Molecular characterisation of the 3’-end of the astrovirus genome. Arch. Virol..

[39] Karlsson E.A., Small C.T., Freiden P., Feeroz M.M., Matsen F.A., San S., Hasan M.K., Wang D., Jones-Engel L., Schultz-Cherry S. (2015). Non-Human primates harbor diverse mammalian and avian astroviruses including those associated with human infections. PLoS Pathog..

[40] Woo P.C.Y., Lau S.K.P., Teng J.L.L., Tsang A.K.L., Joseph S., Xie J., Jose S., Fan R.Y.Y., Wernery U., Yuen K.Y. (2015). A novel astrovirus from dromedaries in the Middle East. J. Gen. Virol..

[41] Donato C., Vijaykrishna D. (2017). The broad host range and genetic diversity of mammalian and avian astroviruses. Viruses.

[42] Fernández-Correa I., Truchado D.A., Gomez-Lucia E., Doménech A., Pérez-Tris J., Schmidt-Chanasit J., Cadar D., Benítez L. (2019). A novel group of avian astroviruses from Neotropical passerine birds broaden the diversity and host range of Astroviridae. Sci. Rep..

[43] Fuller T., Bensch S., Müller I., Novembre J., Pérez-Tris J., Ricklefs R.E., Smith T.B., Waldenström J. (2012). The ecology of emerging infectious diseases in migratory birds: An assessment of the role of climate change and priorities for future research. Ecohealth.

[44] Smyth V.J., Todd D., Trudgett J., Lee A., Welsh M.D. (2012). Capsid protein sequence diversity of chicken astrovirus. Avian Pathol..

[45] McNulty M.S., Connor T.J., McNeilly F., McFerran J.B. (1990). Biological characterisation of avian enteroviruses and enterovirus-like viruses. Avian Pathol..

[46] Todd D., Wilkinson D.S., Jewhurst H.L., Wylie M., Gordon A.W., Adair B.M. (2009). A seroprevalence investigation of chicken astrovirus infections. Avian Pathol..

[47] Kwoka K.T.T., de Rooij M.M.T., Messink A.B., Wouters I.M., Smit L.A.M., Heederik D.J.J., Koopmans M.P.G., Phan M.V.T. (2021). Comparative viral metagenomics from chicken feces and farm dust in the Netherlands. bioRxiv.

[48] Worobey M., Holmes E.C. (1999). Evolutionary aspects of recombination in RNA viruses. J. Gen. Virol..

[49] Shah J.D., Desai P.T., Zhang Y., Scharber S.K., Baller J., Xing Z.S., Cardona C.J. (2016). Development of the intestinal RNA virus community of healthy broiler chickens. PLoS ONE.

[50] Pantin-Jackwood M.J., Day J.M., Jackwood M.W., Spackman E. (2008). Enteric viruses detected by molecular methods in commercial chicken and turkey flocks in the United States between 2005 and 2006. Avian Dis..

[51] Pantin-Jackwood M.J., Spackman E., Day J.M., Rives D. (2007). Periodic monitoring of commercial turkeys for enteric viruses indicates continuous presence of astrovirus and rotavirus on the farms. Avian Dis. Dig..

[52] Yin L., Zhou Q., Mai K., Huang J., Yan Z., Wei X., Shen H., Li Q., Chen L., Zhou Q. (2021). Isolation and characterization of a novel chicken astrovirus in China. Poult. Sci..

[53] Palomino-Tapia V., Mitevski D., Inglis T., van der Meer F., Martin E., Brash M., Provost C., Gagnon C.A., Abdul-Careem M.F. (2020). Chicken Astrovirus (CAstV) molecular studies reveal evidence of multiple past recombination events in sequences originated from clinical samples of White Chick Syndrome (WCS) in Western Canada. Viruses.

[54] Adebiyi A.I., Mcilwaine K., Oluwayelu D.O., Smyth V.J. (2021). Detection and characterization of chicken astrovirus associated with hatchery disease in commercial day-old turkeys in southwestern Nigeria. Arch. Virol..

[55] de Wit J.J., ten Dam G.B., vande Laar J.M.A.M., Biermann Y., Verstegen I., Edens F., Schrier C.C. (2011). Detection and characterization of a new astrovirus in chicken and turkeys with enteric and locomotion disorders. Avian Pathol..

[56] Raji A.A., Ideris A., Bejo M.H., Omar A.R. (2022). Molecular detection, characterisation and serological survey of chicken astrovirus from broiler flocks in Malaysia. Pertanika J. Sci. Technol..

[57] Oluwayelu D.O., Todd D. (2012). Chicken astrovirus infection: Minireview and preliminary serologic evidence of antigenically and genetically distinct chicken astroviruses in Nigerian indigenous chickens. African J. Biomed. Res..

[58] Sharma R.N., Dufayet R., Maufras T., O’Connell K., Tiwari K. (2017). Seroprevalence of antibodies to astrovirus in chickens in Grenada, West Indies. Vet. World.

[59] Xue J., Han T., Xu M., Zhao J., Zhang G. (2017). The first serological investigation of chicken astrovirus infection in China. Biologicals.

[60] Smyth J.A., Connor T.J., McNeilly F., Moffet D.A., Calvert V.M., McNulty M.S. (2007). Studies on the pathogenicity of enterovirus-like viruses in chickens. Avian Pathol..

[61] Zhao W., Wu Z., Yao Y., Qin A., Qian K. (2020). The isolation and molecular characterization of an astrovirus from “yellow” chickens, China. Front. Vet. Sci..

[62] Patel A.K., Pandit R.J., Thakkar J.R., Hinsu A.T., Pandey V.C., Pal J.K., Prajapati K.S., Jakhesara S.J., Joshi C.G. (2017). Complete genome sequence analysis of chicken astrovirus isolate from India. Vet. Res. Commun..

[63] Todd D., Smyth V., Ball N.W., Donnelly B.M., Wylie M., Knowles N.J., Adair B.M. (2009). Identification of chicken enterovirus-like viruses, duck hepatitis virus type 2 and duck hepatitis virus type 3 as astroviruses. Avian Pathol..

[64] Brugère-Picoux J., Vaillancourt J.P., Bouzouaia M., Shivaprasad H.L., Venne D. (2015). Manual of Poultry Diseases.

[65] Rebel J.M.J., Balk F.R.M., Post J., Van Hemert S., Zekarias B., Stockhofe N. (2006). Malabsorption syndrome in broilers. Worlds. Poult. Sci. J..

[66] Rosenberger J., Direction C.S. Update on the Runting-Stunting Syndrome. Ceva Eggs Program Online. http://fs-1.5mpublishing.com/images/ceva/EPO_No3-May2012.pdf.

[67] Nuñez L.F.N., Santander-Parra S.H., Kyriakidis N.C., Astolfi-Ferreira C.S., Buim M.R., De la Torre D., Ferreira A.J.P. (2020). Molecular characterization and determination of relative cytokine expression in naturally infected day-old chicks with chicken astrovirus associated to white chick syndrome. Animals.

[68] Smyth V., Trudgett J., Wylie M., Jewhurst H., Conway B., Welsh M., Kaukonen E., Perko-Mäkelä P. (2013). Chicken astrovirus detected in hatchability problems associated with ‘white chicks’. Vet. Rec..

[69] Olsen D.E. Isolation of a Reovirus-like Agent from Broiler Chicks with Diarrhea and Stunting. Proceedings of the 26th Western Poultry Diseases Conference.

[70] Kouwenhoven B., Vertommen M., Goren E. (1988). Investigations into the role of reovirus in the malabsorption syndrome. Avian Pathol..

[71] Zsak L., Cha R.M., Day J.M. (2013). Chicken parvovirus-induced runting-stunting syndrome in young broilers. Avian Dis..

[72] Smyth V.J., Jewhurst H.L., Wilkinson D.S., Adair B.M., Gordon A.W., Todd D. (2010). Development and evaluation of real-time TaqMan ® RT-PCR assays for the detection of avian nephritis virus and chicken astrovirus in chickens. Avian Pathol..

[73] Mettifogo E., Nuñez L.F.N., Chacón J.L., Santander Parra S.H., Astolfi-Ferreira C.S., Jerez J.A., Jones R.C., Piantino Ferreira A.J. (2014). Emergence of enteric viruses in production chickens is a concern for avian health. Sci. World J..

[74] Spackman E., Day J.M., Pantin-Jackwood M.J. (2010). Astrovirus, reovirus, and rotavirus concomitant infection causes decreased weight gain in broad-breasted white poults. Avian Dis. Dig..

[75] Bayry J., Goudar M.S., Nighot P.K., Kshirsagar S.G., Ladman B.S., Gelb J., Ghalsasi G.R., Kolte G.N. (2005). Erratum: Emergence of a nephropathogenic avian infectious bronchitis virus with a novel genotype in India (Journal of Clinical Microbiology (2005) 43:2 (916–918)). J. Clin. Microbiol..

[76] Sellers H., Linneman E., Icard A.H., Mundt E. (2010). A purified recombinant baculovirus expressed capsid protein of a new astrovirus provides partial protection to runting–stunting syndrome in chickens. Vaccine.

[77] Connor T.J., McNeilly F., McFerran J.B., McNulty M.S. (1987). A survey of avian sera from northern ireland for antibody to avian nephritis virus. Avian Pathol..

[78] Moser L.A., Schultz-Cherry S. (2005). Pathogenesis of astrovirus infection. Viral Immunol..

[79] Rajendra Bulbule N., Sanjay Kapgate S., Madhukar Chawak M. (2014). Infectious causes of gout. Adv. Anim. Vet. Sci..

[80] Nuñez L.F.N., Parra S.H.S., Carranza C., Astolfi-Ferreira C.S., Buim M.R., Ferreira A.J.P. (2016). Detection and molecular characterization of chicken astrovirus associated with chicks that have an unusual condition known as “white chicks” in Brazil. Poult. Sci..

[81] Koci M.D., Kelley L.A., Larsen D., Schultz-Cherry S. (2004). Astrovirus-induced synthesis of nitric oxide contributes to virus control during infection. J. Virol..

[82] Nighot P.K., Moeser A., Ali R.A., Blikslager A.T., Koci M.D. (2010). Astrovirus infection induces sodium malabsorption and redistributes sodium hydrogen exchanger expression. Virology.

[83] Kumar S., Stecher G., Li M., Knyaz C., Tamura K. (2018). MEGA X: Molecular Evolutionary Genetics Analysis across Computing Platforms. Mol. Biol. Evol..

