# Supplementary figures and images for: An Insight into the Molecular Characteristics and Associated Pathology of Chicken Astroviruses
Source: Viruses. 2022 Mar 30;14(4):722. doi: 10.3390/v14040722 (PMC9024793; doi:10.3390/v14040722)

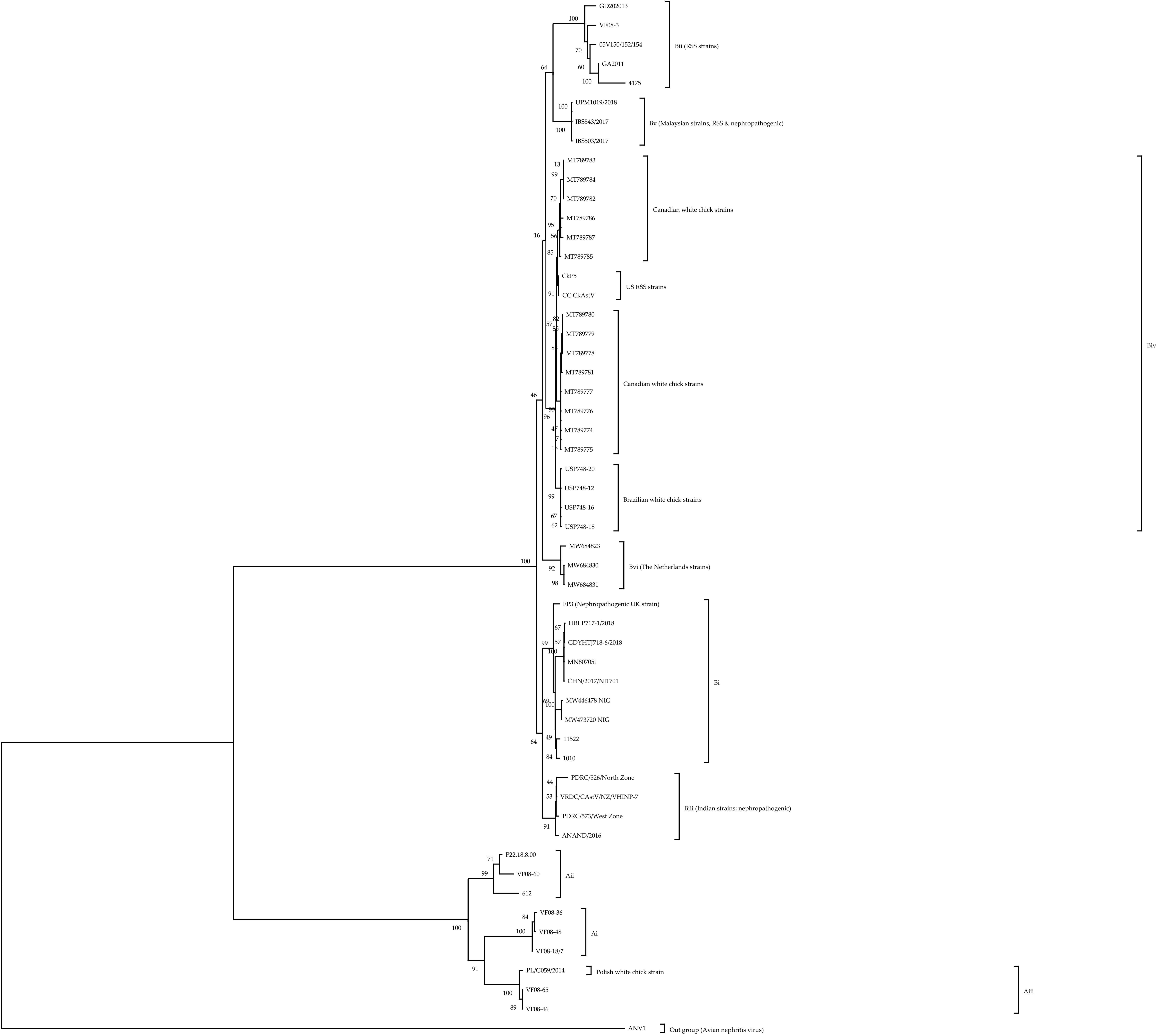

Supplement: Supplementary file 1 [file viruses-14-00722-s001.zip › Figure S1.pdf]
